# Triglyceride Glucose Index and Prognosis of Patients With Ischemic Stroke

**DOI:** 10.3389/fneur.2020.00456

**Published:** 2020-06-10

**Authors:** Yimo Zhou, Yuesong Pan, Hongyi Yan, Yilong Wang, Zixiao Li, Xingquan Zhao, Hao Li, Xia Meng, Chunxue Wang, Liping Liu, Yongjun Wang

**Affiliations:** ^1^Department of Neurology, Beijing Tiantan Hospital, Capital Medical University, Beijing, China; ^2^China National Clinical Research Center for Neurological Diseases, Beijing, China; ^3^Center of Stroke, Beijing Institute for Brain Disorders, Beijing, China; ^4^Beijing Key Laboratory of Translational Medicine for Cerebrovascular Disease, Beijing, China

**Keywords:** insulin resistance, triglyceride glucose index, ischemic stroke, stroke recurrence, prognosis

## Abstract

**Background:** The triglyceride glucose index (TyG index) has been proposed as a simple and credible surrogate marker of insulin resistance. However, it is unclear whether TyG index correlates with adverse clinical outcomes in patients with ischemic stroke. Accordingly, this study aimed to explore the relationship between baseline TyG index and clinical outcomes of ischemic stroke individuals.

**Methods:** We included eligible subjects with ischemic stroke from the China National Stroke Registry II for the current analysis. TyG index was calculated and divided into quartiles to explore the relationship with the outcomes of ischemic stroke. Outcomes included stroke recurrence, all-cause mortality, poor functional outcome at 12 months, and neurologic worsening at discharge. Multivariable Cox regression and logistic regression models were performed to explore the correlation of baseline TyG index with the outcomes.

**Results:** Among the 16,310 patients enrolled in the study, the average age was 64.83 ± 11.9 years, and 63.48% were men. The median TyG index was 8.73 (interquartile range, 8.33–9.21). After adjustment for multiple potential covariates, the fourth quartile of TyG index was associated with an increased risk of stroke recurrence (adjusted HR, 1.32; 95% CI, 1.11–1.57; *P* = 0.002), all-cause mortality (adjusted HR, 1.25; 95%CI, 1.06–1.47; *P* = 0.01) at 12-month follow-up, and neurological worsening (adjusted OR, 1.26; 95% CI, 1.02–1.55; *P* = 0.03) at discharge, but not poor functional outcome compared with the first quartile.

**Conclusion:** TyG index representing insulin resistance was associated with an increased risk of stroke recurrence, all-cause mortality, and neurologic worsening in patients with ischemic stroke.

## Introduction

Stroke is one of the most significant causes of death and disability, with most of the burden in low-income and middle-income countries (1). Improving poststroke outcomes is an urgent issue worldwide. Thus, it is of great importance to identify those stroke patients at high risk for poor outcomes and deliver effective secondary preventions. Insulin resistance (IR) is prevalent in patients with stroke (2). Former studies revealed that IR facilitates the progression of ischemic stroke (IS) through promoting atherosclerosis (3–5), inducing hemodynamic disturbances (6), and accelerating platelet adhesion, activation, and aggregation (7–9), which could trigger stroke recurrence in individuals with IS.

Two large randomized trials have suggested that treatment with pioglitazone, an insulin-sensitizing agent, reduces cardiovascular risk in diabetic patients or nondiabetic patients with IR who were diagnosed with an IS or a transient ischemic attack (TIA) (10, 11). In this sense, IR would be a new target for secondary prevention of stroke for patients with IS or TIA. Recently, a number of studies proved that IR estimated by the homeostasis model assessment of insulin resistance (HOMA-IR) is independently correlated with unfavorable clinical outcomes in patients with IS (12–14). In this regard, the detection of IR in patients after IS could have clinical relevance in secondary prevention of stroke. Although useful in research, the use of HOMA-IR is substantially limited by the need for insulin measurement in clinical practice.

Lately, the triglyceride glucose index (TyG index) has been proposed as a simple surrogate marker of IR (15). Indeed, a number of studies have shown its credibility through assessing by HOMA-IR and hyperinsulinemic-euglycemic clamp test (15–17). Although previous studies have shown that TyG index is associated with carotid atherosclerosis (18), coronary artery calcification (19), coronary artery stenosis (20), and high risk of cardiovascular disease (21), it is not known whether TyG index correlates with adverse clinical outcomes in patients with IS. Moreover, the independent correlation between TyG and prevalent IS in a general population has been uncovered (22). Therefore, this study aimed to explore the relationship between baseline TyG index and clinical outcomes for IS patients through a nationwide prospective registry study.

## Materials and Methods

### Study Cohort and Data Collection

**Figure 1:**
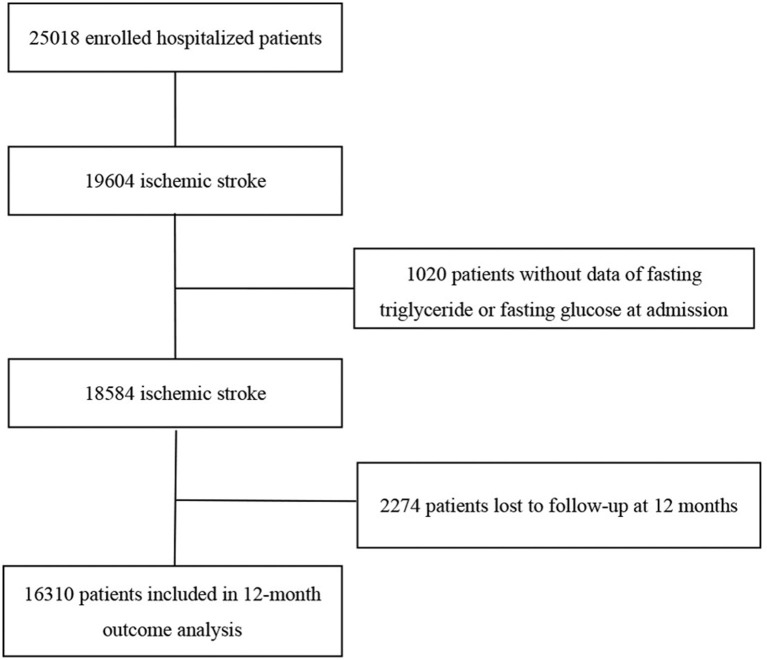
Patient selection flow diagram.

The China National Stroke Registry (CNSR) is a national hospital-based, prospective stroke registry. The registry consists of 3 phases to date. Data collected from September 2007 to August 2008 were used as CNSR phase 1 (CNSR I). This study was conducted on the basis of CNSR phase 2 (CNSR II) which was launched in 2012 aiming at evaluating stroke care delivery in clinical practice (23). CNSR phase 3 has been launched in 2015 and is still in progress (24). The criteria for site selection in CNSR I have been previously published (25). The same criteria were used for selection in CNSR II in order for the hospital characteristics to be in line with those in CNSR I. Patients were eligible if they met the following criteria: age 18 years or older; diagnosis within seven days of the index event of IS, TIA, spontaneous intracerebral hemorrhage, or subarachnoid hemorrhage confirmed by brain imaging; or direct hospital admission from a physician's clinic or emergency department. Among the 25,018 patients in the CNSR II, 19,604 were diagnosed with IS on admission. Ultimately, 16,310 subjects were included in the analysis of the study after excluding patients without data of fasting triglyceride or fasting glucose at admission (*n* = 1,020) and lost to follow-up at 12 months (*n* = 2,274; Figure 1).

Baseline information, including patients' demographics, vascular risk factors, pre-hospital medication history, related laboratory data, and treatment, were collected by trained research coordinators at each participating hospital. Patients were defined as obese according to body mass index (BMI) for the Asian population (BMI ≥ 27.5 kg/m2) provided by the World Health Organization Expert Consultation panel for appropriate BMI. Diabetic patients were classified if either of the following criteria was met: (1) self-reported physician diagnosis of diabetes mellitus; (2) hypoglycemic medications before hospitalization.

The CNSR II study was approved by the Central Institutional Review Board in Beijing Tiantan Hospital, and all written informed consents were obtained from patients or their legally authorized representatives.

### Triglyceride Glucose Index Evaluation

Fasting blood samples were drawn within 24 h of admission, triglyceride and glucose levels were tested by automated enzymatic method at each research center. TyG index was calculated as ln[fasting triglycerides (mg/dl) × fasting glucose (mg/ dl)/2] (16). All measurements were performed by trained laboratory personnel blinded to subjects' clinical situations.

### Patient Follow-up and Outcome Evaluation

Patients enrolled were followed up by telephone interview at 12-month after disease onset according to the protocol of CSNR II study. Clinical outcomes, including recurrent stroke, all-cause mortality, and modified Rankin Scale (mRS) score, were obtained by trained research coordinators who were blinded to subjects' baseline characteristics. The recurrent stroke included IS, intracranial hemorrhage, and subarachnoid hemorrhage. Poor functional outcome was defined as mRS scores of 3 to 6. The poststroke clinical course during hospitalization was also evaluated using neurologic worsening, which was defined as a ≥2-point increase in National Institutes of Health Stroke Scale (NIHSS) score hospitalization compared with the NIHSS score on admission (26).

### Statistical Analysis

Continuous variables are described as means ± standard deviations (SDs) or medians with interquartile range (IQRs) and categorical variables as proportions. The demographic and clinical characteristics stratified by TyG index quartiles were compared by using the χ2 test for categorical variables and analysis of variance or Kruskal–Wallis test for continuous variables, respectively. In the main analysis of the study, the TyG index was examined as quartiles to explore the relationship with the outcomes of IS. Taking the lowest quantiles of TyG index as the reference, we performed Cox regression models to explore the correlation of the categories of TyG index with stroke recurrence and all-cause mortality and logistic regression models for its relationship with poor functional outcome at 12 months and neurologic worsening during hospitalization. Adjusted hazard ratios (HR) or odds ratios (OR) with their 95% confidence intervals (CIs) were estimated in each regression model. Multivariable models were adjusted for characteristics that were significantly different at baseline. Besides, to further evaluate the pattern and magnitude of the relationship between TyG index on a continuous scale and the risk of stroke recurrence, all-cause mortality, poor functional outcome, and neurologic worsening, we used a logistic regression model or Cox regression model with restricted cubic splines for TyG index. In the sensitivity analysis, we excluded patients with diabetes or obesity. The TyG index was evaluated as continuous to investigate a potential dose-response relationship with the outcomes of IS.

All statistical analyses were conducted with SAS software version 9.4 (SAS Institute Inc., Cary, NC). All P values were 2-sided, with *P* values < 0.05 considered significant.

## Results

### Baseline Characteristics

The included patients in the study were stratified into four groups based on their TyG index levels. The baseline characteristics of the patients according to the TyG index quartiles are demonstrated in Table 1. The average age of the patients was 64.83 ± 11.9 years, and 63.48% were men. The median TyG index was 8.73 (IQR, 8.33–9.21). The patients with higher TyG index tended to have higher systolic and diastolic blood pressure and lower frequency of smoking and were more likely to receive antihypertensive drugs and hypoglycemia drugs before admission. Besides, significant differences in the history of hypertension, atrial fibrillation, diabetes mellitus, and previous stroke were also observed among groups. Parameters related to glucose metabolism (BMI, triglyceride, and fasting blood pressure at admission) increased as the TyG index increased.

**Table 1:** Baseline characteristics according to TyG index quartiles.

	**Total patients**	**TyG index**				
		**Q1 (*n* = 4078)**	**Q2 (*n* = 4077)**	**Q3 (*n* = 4078)**	**Q4 (*n* = 4077)**	***P*-value**
Sex (male) (%)	10,353 (63.5)	2,810 (68.9)	2,632 (64.6)	2,544 (62.4)	2,367 (58.1)	**<0.0001**
Age (years)	64.8 ± 11.9	67.5 ± 12.2	65.4 ± 11.8	64.0 ± 11.8	62.5 ± 11.3	**<0.0001**
NIHSS	4 (2–7)	4 (2–7)	4 (2–7)	4 (2–7)	4 (2–7)	0.13
IV thrombolysis (%)	890 (5.6)	218 (5.5)	229 (5.8)	215 (5.4)	228 (5.7)	0.88
SBP (mmHg)	149.2 ± 23.0	147.8 ± 23.6	148.8 ± 22.3	149.3 ± 22.3	151.0 ± 23.1	**<0.0001**
DBP (mmHg)	87.44 ± 13.4	85.9 ± 13.6	87.4 ± 13.4	88.0 ± 13.3	88.6 ± 13.2	**<0.0001**
Previous or current smoker (%)	7246 (44.4)	1921 (47.1)	1824 (44.7)	1780 (43.7)	1721 (42.2)	**<0.0001**
**Medical History**						
History of hypertension (%)	10593 (65.0)	2330 (57.1)	2633 (64.6)	2773 (68.0)	2857 (70.1)	**<0.0001**
History of AF (%)	1143 (7.0)	383 (9.4)	274 (6.7)	263 (6.5)	223 (5.5)	**<0.0001**
History of MI (%)	289 (2.4)	90 (2.2)	104 (2.6)	102 (2.5)	93 (2.9)	0.69
History of DM (%)	3408 (20.9)	277 (6.8)	501 (12.3)	855 (21.0)	1775 (43.5)	**<0.0001**
History of previous stroke (%)	5367 (32.9)	1386 (34.0)	1354 (33.2)	1372 (33.6)	1255 (30.8)	0.0086
**Pre-Hospital Mediation History**						
Antiplatelet drugs (%)	3191 (19.6)	786 (19.3)	822 (20.2)	769 (18.9)	814 (20.0)	0.41
Anticoagulation drugs (%)	168 (1.0)	50 (1.2)	49 (1.2)	36 (0.9)	33 (0.8)	0.14
Antihypertensive drugs (%)	7324 (44.9)	1564 (38.4)	1821 (44.7)	1924 (47.2)	2015 (49.4)	**<0.0001**
Lipid-lowing drugs (%)	1103 (6.8)	263 (6.5)	273 (6.7)	278 (6.8)	289 (7.1)	0.71
Hypoglycemic drugs (%)	2604 (16.0)	217 (5.3)	373 (9.2)	661 (16.2)	1353 (33.2)	**<0.0001**
**Glucose Metabolism**						
BMI (kg/m2)	24.2 ± 3.6	23.2 ± 3.5	24.0 ± 3.4	24.6 ± 3.4	24.9 ± 3.8	**<0.0001**
TG (mg/dL)	119.5 (85.9−174.4)	70.0 (58.4−82.3)	106.3 (92.9−120.4)	147.9 (124.0−172.7)	228.5 (177.1−301.9)	**<0.0001**
FBG (mg/dL)	114.1 ± 46.9	89.8 ± 16.3	98,7 ± 20.7	111.7 ± 31.1	156.2 ± 67.4	**<0.0001**

*Data are the mean ± standard deviation, number (percentage), or median (interquartile range); TyG index, triglyceride glucose index; NIHSS, National Institutes of Health Stroke Scale; IV, intravenous; SBP, systolic blood pressure; DBP, diastolic blood pressure; AF, atrial fibrillation; MI, myocardial infarction; DM, diabetes mellitus; BMI, body mass index; TG, Triglyceride; FBG, Fasting blood glucose; Q1–Q4, TyG index quartiles; Statistically significant P values are shown in bold*.

### 12-Month Clinical Outcomes According to TyG Index

Table 2 shows the association between clinical outcomes of IS and TyG index quartiles. Adjusted hazard ratios/odds ratios with 95% confidence intervals of the TyG index for clinical outcomes are presented in Table 2. The relationship between the TyG index and the clinical outcomes were explored by categorizing the TyG index into quartiles using the first quartile as the reference. After adjusting for multivariate, it was found that patients with a TyG index of Q4 (≥9.21) were associated with increased risk of stroke recurrence and all-cause mortality at 12-month follow-up (adjusted HR, 1.32; 95% CI, 1.11–1.57; *P* = 0.002 and adjusted HR, 1.25; 95%CI, 1.06–1.47; *P* = 0.01, respectively) compared with patients with a TyG index of Q1 (≤ 8.33). After adjustment for age, sex, and potential confounders in a logistic regression model, the patients with a TyG index of Q4 were associated with an elevated risk of neurological worsening at discharge (adjusted OR, 1.26; 95% CI, 1.02–1.55; *P* = 0.03) compared with patients with a TyG index of Q1. However, no significant association was observed between TyG index and poor functional outcomes at 12-month follow-up in patients with IS.

**Table 2:** Adjusted odds ratios/hazard ratios of TyG index quartiles for clinical outcomes.

				**Age and sex- adjusted**		**Multivariable- adjusted**	
	**TyG index group**	***n***	**Events (%)**	**OR/HR (95%CI)**	***P***	**OR/HR (95%CI)**	***P***
Stroke recurrence	Q1 (≤ 8.33)	4078	282 (6.92)	1.00		1.00	
	Q2 (8.34−8.73)	4077	270 (6.62)	1.00 (0.85-1.18)	0.10	1.00 (0.84-1.78)	0.96
	Q3 (8.74−9.20)	4078	290 (7.11)	1.11 (0.94-1.31)	0.21	1.08 (0.91-1.28)	0.36
	Q4 (≥9.21)	4077	350 (8.58)	1.41 (1.20-1.65)	**<0.0001**	1.32 (1.11-1.57)	**0.002**
All-cause mortality	Q1 (≤ 8.33)	4078	385 (9.44)	1.00		1.00	
	Q2 (8.34−8.73)	4077	340 (8.34)	1.02 (0.89-1.19)	0.75	1.07 (0.92-1.24)	0.39
	Q3 (8.74−9.20)	4078	298 (7.31)	0.98 (0.86-1.15)	0.84	1.01 (0.86-1.18)	0.91
	Q4 (≥9.21)	4077	330 (8.09)	1.26 (1.08-1.46)	**0.003**	1.25 (1.06-1.47)	**0.008**
Poor functional outcome	Q1 (≤ 8.33)	4078	1013 (24.84)	1.00		1.00	
	Q2 (8.34−8.73)	4077	916 (22.47)	0.99 (0.89-1.10)	0.81	0.99 (0.88-1.10)	0.71
	Q3 (8.74−9.20)	4078	862 (21.14)	0.99 (0.89-1.11)	0.88	1.04 (0.93-1.16)	0.54
	Q4 (≥9.21)	4077	862 (21.14)	1.10 (0.99-1.23)	0.09	0.99 (0.88-1.12)	0.90
Neurologic worsening	Q1 (≤8.33)	3905	216 (5.53)	1.00		1.00	
	Q2 (8.34−8.73)	3873	215 (5.55)	1.05 (0.86-1.27)	0.65	1.06 (0.87-1.29)	0.57
	Q3 (8.74−9.20)	3861	233 (6.03)	1.17 (0.97-1.42)	0.11	1.17 (0.96-1.43)	0.11
	Q4 (≥9.21)	3872	244 (6.30)	1.27 (1.05-1.54)	**0.02**	1.26 (1.02-1.55)	**0.03**

*TyG index, triglyceride glucose index; Q1–Q4, TyG index quartiles; CI, confidence interval; HR, hazard ratio; OR, odds ratio; HR for stroke recurrence and all-cause mortality, while OR for poor functional outcome, and neurologic worsening; Statistically significant P values are shown in bold; The multivariable-adjusted model included age, sex, body mass index, systolic blood pressure, diastolic blood pressure, previous or current smoker, history of hypertension, history of atrial fibrillation, history of diabetes mellitus, history of previous stroke, pre-hospital medication history of antihypertensive drugs and hypoglycemic drugs*.

**Figure 2:**
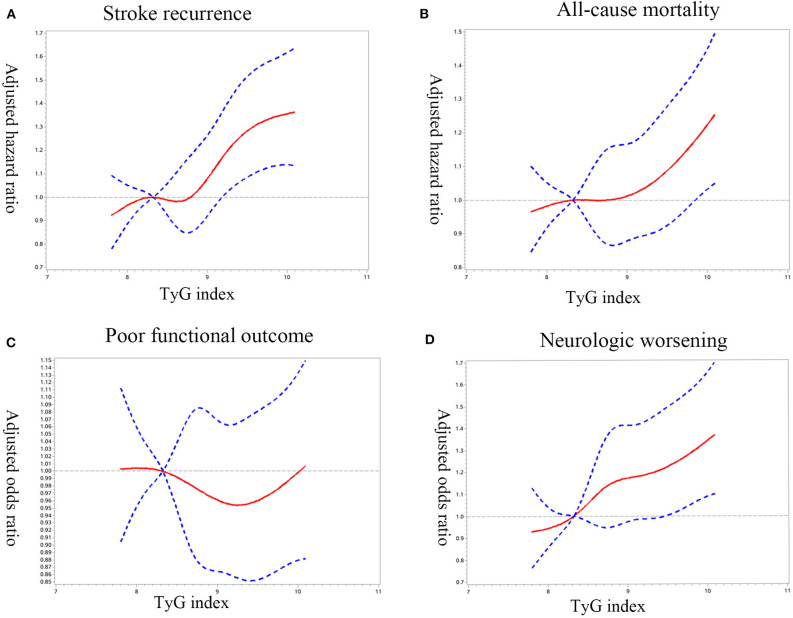
Adjusted hazard ratios/odds ratio of ischemic stroke prognosis according to triglyceride glucose index (TyG index) on admission. Adjusted hazard ratios/odds ratio of **(A)** stroke recurrence, **(B)** all-cause mortality, **(C)** poor functional outcome, and **(D)** neurologic worsening according to TyG index. HR/ORs were obtained by restricted cubic spline Cox/logistic regression after adjustment for confounding factors, with the first quartile of TyG index as reference. The solid red line indicates the adjusted hazard ratio/odds ratio and the dashed blue lines the 95% confidence interval bands.

Cox/logistic regression analyses with restricted cubic spline further demonstrated that higher baseline TyG index levels significantly associated with increased risk of recurrent stroke (Figure 2A), all-cause mortality (Figure 2B), and neurological worsening (Figure 2D); While no significant association was found between TyG index and poor functional outcomes (Figure 2C).

### Sensitivity Analysis

In the sensitivity analyses of continuous TyG index, the significant association was observed between TyG index (per unit) and stroke recurrence (adjusted HR, 1.19; 95% CI, 1.09–1.30; *P* < 0.0001) and between all-cause mortality (adjusted HR, 1.12; 95% CI, 1.02–1.22; *P* = 0.01) and neurological worsening (adjusted OR, 1.19; 95% CI, 1.07–1.32; *P* = 0.001) after adjusting for multiple variables (Table 3).

**Table 3:** Adjusted odds ratios/hazard ratios of TyG index as continuous variable (per unit) for clinical outcomes.

			**Age and sex—adjusted**		**Multivariable—adjusted**	
	***n***	**Events (%)**	**OR/HR (95%CI)**	***P***	**OR/HR (95%CI)**	***P***
Stroke recurrence	16310	1192 (7.31)	1.23 (1.14-1.33)	**<0.0001**	1.19 (1.09-1.30)	**<0.0001**
All-cause mortality	16310	1353 (8.30)	1.13 (1.04-1.22)	**0.003**	1.12 (1.02-1.22)	**0.01**
Poor functional outcomes	16310	3655 (22.41)	1.06 (1.00-1.12)	**0.045**	1.00 (0.94-1.06)	0.93
Neurologic worsening	15511	908 (5.85)	1.19 (1.08-1.31)	**0.0004**	1.19 (1.07-1.32)	**0.001**

*TyG index, triglyceride glucose index; Q1–Q4, TyG index quartiles; CI, confidence interval; HR, hazard ratio; OR, odds ratio; HR for stroke recurrence and all-cause mortality, while OR for poor functional outcome, and neurologic worsening; Statistically significant P values are shown in bold; The multivariable-adjusted model included age, sex, body mass index, systolic blood pressure, diastolic blood pressure, previous or current smoker, history of hypertension, history of atrial fibrillation, history of diabetes mellitus, history of previous stroke, pre-hospital medication history of antihypertensive drugs and hypoglycemic drugs*.

Finally, we assessed the aforementioned associations after excluding patients with obesity or diabetes. The multivariable-adjusted HR/OR of the above-described clinical outcomes showed a similar association for the TyG index even in nonobese patients (Supplementary Table 1) and nondiabetic patients (Supplementary Table 2). However, no significant association was observed between TyG index and all-cause mortality in nondiabetic patients.

## Discussion

The major findings of the study are as follows. We found that IR, estimated by TyG index, was significantly associated with an increased risk of 12-month stroke recurrence, all-cause mortality, and neurologic worsening during hospitalization without accelerating 12-month poor functional outcome in patients suffered from IS.

Various pathophysiological pathways may underlie the association between IR and IS. Firstly, IR has been proposed to facilitate the pathogenesis of atherosclerosis (27). IR enhances the pathologic process of vascular endothelia cell, macrophages, and smooth muscle cells via inflammation, which contributes to the progression of atherosclerosis (28–30). Moreover, atherogenic impact through atherogenic dyslipidemia and the impaired fibrinolysis could be exerted by IR (31–33). Secondly, a body of researches has revealed that IS plays an important role in platelet adhesion, activation, and aggregation, which are associated with vessel occlusion and involved in IS incidents (34–37). Furthermore, Lundstrom et al. found that a high level of IR was a pre-requisite for high on-treatment platelet reactivity in minor IS or TIA patients prescribed clopidogrel as secondary prevention, which may influence the efficacy of antiplatelet treatment and lead to poor prognosis (38). Thirdly, IR causes hemodynamic disturbance. An attenuate cerebrovascular reserve (CVR) has been observed in patients with diabetes or non-diabetic IR (39, 40). Previous studies discovered that IR is involved in CVR through Bayliss effect, and chemical, neuronal, and metabolic mechanisms (41–44). Last, IR enlarges the role of modifiable risk factors in IS, such as hypertension, diabetes, dyslipidemia, and cigarette smoking (45–47). It may accelerate the progression of atherosclerosis by modifying the risk factors through inflammation and oxidative stress mechanisms and damage the cerebral metabolism (48, 49).

To date, previous studies mostly used HOMA-IR for the assessment of IR to evaluate the correlation between IR and clinical outcomes in patients with IS (13, 50, 51). However, it is usually impractical in clinical practice and large-scale research because of the need to measure insulin level.

In recent years, TyG index has been proposed to estimate insulin sensitivity by simply assessing circulating triglyceride and glucose concentration in routine clinical practice (15). Several potential mechanisms have been suggested to explain the correlation between TyG index and IR. It has been well established that hyperglycemia and dyslipidemia are two basic hallmarks of IR. IR may play a vital role in the development of hyperglycemia and dyslipidemia, which can further aggravate IR. As for whole-body glucose homeostasis, the expression and activity of glucose transporter are modulated by IR in multiple tissues, promoting glucose accumulation in circulation and forming hyperglycemia (52). Moreover, hyperglycemia may, in turn, impair insulin sensitivity, resulting in a vicious circle toward IR (53). With regard to lipid profiles, IR result in excessive release of free fatty acid into circulation by unstrained lipolysis (54, 55). The increased flux of free fatty acids increases hepatic triglyceride synthesis in the liver and release in plasma, which gives rise to hypertriglyceridemia and subsequent metabolic syndrome (54, 55). Meanwhile, increased circulating fatty acids may also inhibit insulin activity of glucose uptake in peripheral tissues and lipolysis, leading to exacerbation of IR and the development of another vicious circle between IR and dyslipidemia (56). Therefore, hypertriglyceridemia is considered as an excellent reflection of the IR condition (54). Overall, TyG index, the product of fasting triglyceride and glucose, could be used as a surrogate marker for the assessment of IR.

Although hyperglycemia and dyslipidemia underlie the mechanism of IR as we mentioned above, it may be inappropriate applying fasting glucose or triglycerides alone as surrogate estimates for IR. It is known that glucose metabolism is maintained within the normal range as long as hyperinsulinemia is efficient to surmount IR (57). The Metabolic Syndrome in Men study demonstrated that insulin sensitivity was already decreased substantially within the normal range of fasting plasma glucose (58). Therefore, fasting glucose alone may not be an accurate estimate for IR. As for triglyceride, a study conducted in a large Chinese population made a head-to-head comparison between lipid (including triglyceride), apolipoprotein measures, lipid ratios, lipid accumulation product, visceral adiposity index, and TyG index as predictors of IR. TyG index appeared to be more closely associated with IR than any other lipid-related variables studied, and proved to be the best discriminator in identifying IR (59). Accordingly, we considered TyG index a comprehensive marker for IR because both glucotoxicity and lipotoxicity are critical mechanisms in modulation of IR. In addition, a number of studies have identified the great correlations between TyG and hyperinsulinemic-euglycemic clamp test or HOMA-IR in different populations (16, 17, 60, 61). Thus, the TyG index is being considered as a credible and straightforward surrogate marker of IR for clinical practice.

The association between IR and incidence of IS has been well investigated in general population (62–64). However, whether IR correlated with clinical prognosis of individuals with IS is still a matter of debate. Therefore, we conducted our study to investigate the correlation in a large-scale, prospective cohort. In accordance with previous studies from the ACROSS-China registry (Abnormal Glucose Regulation in Patients with Acute Stroke Across China) (12, 65), the results in the main analysis of the study support that IR significantly increases the risk of stroke recurrence, all-cause mortality during the 12-month follow-up. The relationship between IR and neurologic worsening tested in the study is also in line with the result reported by Ago et al. (13). However, our study suggests that IR is not predictive of poor functional outcome, which is contrary to some study findings (12–14, 65). The different findings might have been caused by differences in designs of the studies, follow-up times, diagnostic methods, and ethnicities studied. In addition, there is no previous study of TyG index in the IS population, and the TyG index could be different from other markers of IR measurement.

This study, to the best of our knowledge, is the first study to associate the TyG index with clinical outcomes in patients with IS. Our study added evidence of the correlation between IR and prognosis of patients with IS based on a large-scale Chinese population. Furthermore, the IRIS study has provided evidence that treatment of insulin-sensitizing agent is effective in secondary prevention for individuals suffering from IS and IR. Thus, our study demonstrated that the TyG index would be an easily obtainable marker for risk stratification in IS patients during daily clinical practice.

The present study had several limitations. First, fasting triglyceride and glucose levels were tested at each study center. Thus, measurement errors derived from different analytic systems cannot be completely excluded. However, the testing results would be comparable because the measurement of triglyceride and glucose in all centers was based on the recommendation of International Federation of Clinical Chemistry and Laboratory Medicine (2011). Second, 3,294 (16.8%) patients were excluded from the study because of missing data or loss to follow-up at 12-month. The patients excluded were with higher NIHSS at admission, lower rate for antiplatelet treatment before the index event, and lower BMI, compared with the subjects included (Supplementary Table 3). Thus, a selective bias could not be avoided. Third, while recent studies indicated that imaging and biological markers are determinant factors to predict the prognosis of stroke (66, 67), these variables were not collected and analyzed in our study. Therefore, there is potential bias due to residual confounding from these unmeasured variables. Fourth, our study included acute IS patients within seven days from the onset of symptoms, and blood samples usually were not taken directly after falling ill in IS. As a result, the TyG index would reflect stress hyperglycemia to a large extent; additionally, fasting glucose and triglyceride level were influenced by various interventions given before admission which could be impossible to manage thoroughly in the study. Fifth, different quality of stroke care may also cause potential bias, allowing the fact that this study derived data from a nationwide cohort including hospitals with different geographic region, teaching status, hospital beds, and annual stroke discharges. Last, because our cohort included exclusively Chinese patients, the generalizability of the findings should be further validated in other ethnic populations. It is noticeable that this study only demonstrated the relationship between TyG index and adverse clinical outcomes in AIS patients, and further works are still needed to compare the predictive values of fasting glucose and triglyceride alone with TyG index in the future.

## Conclusions

In this nationwide, large-scale stroke registry, higher TyG index representing IR was associated with an increased risk of stroke recurrence, all-cause mortality, and neurologic worsening in patients with IS. These findings may provide useful information for researchers interested in the fields of TyG index and stroke risk prediction not only for studies but also for clinical and public health applications.

## Data Availability Statement

The datasets generated for this study are available on request to the corresponding author.

## Ethics Statement

The studies involving human participants were reviewed and approved by the Central Institutional Review Board in Beijing Tiantan Hospital. The patients/participants provided their written informed consent to participate in this study.

## Author Contributions

YZ, YP, and YoW conceptualize and design the study. YiW, XZ, CW, and LL assisted with data acquisition and interpretation. ZL and XM coordinated the study. YP, HY, and HL conducted the statistical analysis. YZ prepared the manuscript. YoW is the guarantor for this paper. All authors read and approved the final manuscript.

## Conflict of Interest

The authors declare that the research was conducted in the absence of any commercial or financial relationships that could be construed as a potential conflict of interest.

## Nomenclature

TyG: triglyceride glucose

IR: insulin resistance

IS: ischemic stroke

TIA: transient ischemic attack

HOMA-IR: homeostasis model assessment of insulin resistance

CNSR: China National Stroke Registry

BMI: body mass index

mRS: modified Rankin Scale

NIHSS: National Institutes of Health Stroke Scale

SD: standard deviations

IQR: interquartile range

HR: hazard ratio

OR: odds ratio

CI: confident interval

IV: intravenous

CVR: cerebrovascular reserve
