# Supplementary material for: Triglyceride Glucose Index and Prognosis of Patients With Ischemic Stroke
Source: Front Neurol. 2020 Jun 10;11:456. doi: 10.3389/fneur.2020.00456 (PMC7297915; doi:10.3389/fneur.2020.00456)
Supplement: Supplementary file 1 [file Data_Sheet_1.docx]

Supplementary Material

# Supplementary Figures and Tables

Supplementary Table 1: Adjusted odds ratios/hazard ratios of TyG index quartiles for clinical outcomes in non-obesity patients (n=14291)

|  |  |  |  | Age and sex-adjusted | | Multivariable- adjusted | |
| --- | --- | --- | --- | --- | --- | --- | --- |
|  | TyG index group | n | Events (%) | OR/HR (95%CI) | P | OR/HR (95%CI) | P |
| Stroke recurrence | Q1 (≤ 8.30) | 3573 | 239 (6.69) | 1.00 | | 1.00 | |
|  | Q2 (8.31 -8.70) | 3578 | 233 (6.51) | 1.03 (0.86-1.23） | 0.79 | 1.02 (0.85-1.23) | 0.81 |
|  | Q3 (8.71 -9.17) | 3568 | 253 (7.09) | 1.15 (0.96-1.38） | 0.12 | 1.12 (0.93-1.34) | 0.23 |
|  | Q4 (≥9.19) | 3572 | 311 (8.71) | 1.50 (1.26-1.78) | **<0.0001** | 1.41 (1.18-1.70) | **0.0002** |
| All-cause mortality | Q1 (≤ 8.30) | 3573 | 337 (9.43) | 1.00 | | 1.00 | |
|  | Q2 (8.31 -8.70) | 3578 | 302 (8.44) | 1.04 (0.89-1.22) | 0.62 | 1.10 (0.94-1.29) | 0.24 |
|  | Q3 (8.71 -9.17) | 3568 | 280 (7.85) | 1.05 (0.89-1.23) | 0.58 | 1.08 (0.92-1.28) | 0.34 |
|  | Q4 (≥9.19) | 3572 | 289 (8.09) | 1.26 (1.08-1.48) | **0.005** | 1.28 (1.08-1.52) | **0.005** |
| Poor functional outcome | Q1 (≤ 8.30) | 3573 | 896 (25.08) | 1.00 |  | 1.00 |  |
|  | Q2 (8.31 -8.70) | 3578 | 789 (22.05) | 0.94 (0.84-1.06) | 0.31 | 0.95 (0.84-1.06) | 0.35 |
|  | Q3 (8.71 -9.17) | 3568 | 789 (22.11) | 1.01 (0.90-1.14) | 0.83 | 0.99 (0.87-1.11) | 0.82 |
|  | Q4 (≥9.19) | 3572 | 758 (21.22) | 1.07 (0.95-1.21) | 0.24 | 0.97 (0.86-1.11) | 0.68 |
| Neurologic worsening | Q1 (≤ 8.30) | 3421 | 182 (5.32) | 1.00 | | 1.00 | |
|  | Q2 (8.31 -8.70) | 3403 | 189 (5.56) | 1.09 (0.88-1.34) | 0.43 | 1.12 (0.91-1.39) | 0.29 |
|  | Q3 (8.71 -9.17) | 3373 | 190 (5.63) | 1.13 (0.91-1.40) | 0.26 | 1.15 (0.93-1.43) | 0.19 |
|  | Q4 (≥9.19) | 3397 | 219 (6.45) | 1.36 (1.10-1.67) | **0.004** | 1.38 (1.11-1.73) | **0.004** |

TyG index Triglyceride glucose index, Q1-Q4 TyG index quartiles, CI confidence interval, HR hazard ratio, OR odds ratio; HR for stroke recurrence and all-cause mortality, while OR for poor functional outcome, and neurologic worsening; Statistically significant P values are shown in bold; The multivariable-adjusted model included age, sex, body mass index, systolic blood pressure, diastolic blood pressure, previous or current smoker, history of hypertension, history of atrial fibrillation, history of diabetes mellitus, history of previous stroke, pre-hospital medication history of antihypertensive drugs and hypoglycemic drugs.

Supplementary Table 2: Adjusted odds ratios/hazard ratios of TyG index quartiles for clinical outcomes in non-diabetes patients (n=12902)

|  |  |  |  | Age and sex-adjusted | | Multivariable- adjusted | |
| --- | --- | --- | --- | --- | --- | --- | --- |
|  | TyG index group | n | Events (%) | OR/HR (95%CI) | P | OR/HR (95%CI) | P |
| Stroke recurrence | Q1 (≤ 8.25) | 3225 | 219 (6.79) | 1.00 | | 1.00 | |
|  | Q2 (8.26 -8.68) | 3217 | 208 (6.47) | 0.99 (0.82-1.20） | 0.94 | 1.00 (0.82-1.21) | 0.97 |
|  | Q3 (8.69 -9.04) | 3234 | 209 (6.46) | 1.04 (0.86-1.25） | 0.72 | 1.04 (0.86-1.26) | 0.69 |
|  | Q4 (≥9.05) | 3226 | 241 (7.47) | 1.27 (1.05-1.53) | **0.01** | 1.28 (1.06-1.55) | **0.01** |
| All-cause mortality | Q1 (≤ 8.25) | 3225 | 308 (9.55) | 1.00 | | 1.00 | |
|  | Q2 (8.26 -8.68) | 3217 | 265 (8.24) | 0.99 (0.84-1.16) | 0.87 | 1.05 (0.89-1.23) | 0.60 |
|  | Q3 (8.69 -9.04) | 3234 | 242 (7.48) | 1.01 (0.85-1.20) | 0.89 | 1.08 (0.91-1.28) | 0.38 |
|  | Q4 (≥9.05) | 3226 | 206 (6.39) | 1.01 (0.84-1.21) | 0.93 | 1.10 (0.91-1.32) | 0.32 |
| Poor functional outcome | Q1 (≤ 8.25) | 3225 | 792 (24.56) | 1.00 |  | 1.00 | |
|  | Q2 (8.26 -8.68) | 3217 | 716 (22.26) | 0.98 (0.87-1.10) | 0.71 | 0.98 (0.87-1.11) | 0.79 |
|  | Q3 (8.69 -9.04) | 3234 | 670 (20.72) | 0.99 (0.97-1.12) | 0.85 | 1.00 (0.88-1.14) | 0.98 |
|  | Q4 (≥9.05) | 3226 | 564 (17.48) | 0.90 (0.79-1.03) | 0.13 | 0.92 (0.80-1.05) | 0.21 |
| Neurologic worsening | Q1 (≤ 8.25) | 3095 | 168 (5.43) | 1.00 | | 1.00 | |
|  | Q2 (8.26 -8.68) | 3058 | 172 (5.62) | 1.08 (0.87-1.35) | 0.48 | 1.10 (0.88-1.37) | 0.40 |
|  | Q3 (8.69 -9.04) | 3084 | 184 (5.97) | 1.20 (0.96-1.49) | 0.11 | 1.20 (0.96-1.50) | 0.10 |
|  | Q4 (≥9.05) | 3075 | 185 (6.02) | 1.26 (1.01-1.57) | **0.04** | 1.27 (1.01-1.59) | **0.04** |

TyG index Triglyceride glucose index, Q1-Q4 TyG index quartiles, CI confidence interval, HR hazard ratio, OR odds ratio; HR for stroke recurrence and all-cause mortality, while OR for poor functional outcome, and neurologic worsening; Statistically significant P values are shown in bold; The multivariable-adjusted model included age, sex, body mass index, systolic blood pressure, diastolic blood pressure, previous or current smoker, history of hypertension, history of atrial fibrillation, history of diabetes mellitus, history of previous stroke, pre-hospital medication history of antihypertensive drugs and hypoglycemic drugs.

Supplementary Table 3: Baseline characteristics between the study patients and those excluded

|  | Patients included  (n=16310) | Patients excluded  (n=3294) | P value |
| --- | --- | --- | --- |
|  |  |  |  |
| Sex(male), n (%) | 5957 (36.52) | 1210 (36.73) | 0.820 |
| Age, (SD)y | 64.83±11.92 | 64.85±12.29 | 0.644 |
| NIHSS at admission (IQR) | 4 (2-7) | 4 (2-8) | **<0.001** |
| IV thrombolysis, n (%) | 890 (5.59) | 196 (6.08) | 0.267 |
| Systolic blood pressure, (SD) mmHg | 149.20±23.0 | 148.92±22.89 | 0.459 |
| Diastolic blood pressure, (SD) mmHg | 87.43±13.39 | 87.12±13.25 | 0.207 |
| Previous or current smoker, n (%) | 7246 (44.43) | 1427 (43.32) | 0.244 |
| **Medical history** |  |  |  |
| History of hypertension, n (%) | 10593 (64.95) | 2104 (63.87) | 0.239 |
| History of atrial fibrillation, n (%) | 1143 (7.01) | 239 (7.26) | 0.613 |
| History of myocardial infarction, n (%) | 389 (2.39) | 90 (2.73) | 0.239 |
| History of diabetes mellitus, n (%) | 3408 (20.90) | 652 (19.79) | 0.155 |
| History of previous stroke, n (%) | 5367 (32.91) | 1109 (33.67) | 0.397 |
| **Pre-hospital mediation history** |  |  |  |
| Antiplatelet drugs (%) | 14493 (93.75) | 2819 (91.23) | **<0.001** |
| Anticoagulation drugs (%) | 168 (1.03) | 40 (1.21) | 0.346 |
| Antihypertensive drugs (%) | 7324 (44.90) | 1451 (44.05) | 0.368 |
| Lipid-lowing drugs (%) | 1103 (6.76) | 248 (7.53) | 0.114 |
| Hypoglycemic drugs (%) | 2604 (15.97) | 511 (15.51) | 0.517 |
| **Glucose metabolism** |  |  |  |
| BMI, (SD)kg/m2 | 24.2±3.58 | 23.84±3.61 | **<0.001** |
| Triglyceride at admission, (IQR)mg/dL | 119.54 (85.89-174.43) | 119.54 (85.89-175.32) | 0.674 |
| Fasting blood glucose, (SD)mg/dL | 114.12±46.90 | 115.46±49.34 | 0.623 |

Data are the mean ± Standard deviation, number (percentage), or median (interquartile range); TyG index Triglyceride glucose index, NIHSS National Institutes of Health Stroke Scale, IV intravenous, ﻿SBP systolic blood pressure, DBP diastolic blood pressure, AF atrial fibrillation, MI myocardial infarction, DM diabetes mellitus, BMI body mass index, TG Triglyceride, FBG Fasting blood glucose at admission, Q1-Q4 indicate TyG index quartiles; Statistically significant P values are shown in bold.
